# Cervical small cell carcinoma frequently presented in multiple high risk HPV infection and often associated with other type of epithelial tumors

**DOI:** 10.1186/s13000-018-0709-9

**Published:** 2018-05-22

**Authors:** Peifeng Li, Jing Ma, Xiumin Zhang, Yong Guo, Yixiong Liu, Xia Li, Danhui Zhao, Zhe Wang

**Affiliations:** State Key Laboratory of Cancer Biology, Department of Pathology, Xijing Hospital and School of Basic Medicine, The Fourth Military Medical University, Changle West Road #169, Xi’an, 710032 Shaan Xi Province China

**Keywords:** Human papillomavirus, p16, p53, Small cell carcinoma, Uterine cervix

## Abstract

**Background:**

Small cell carcinoma of the uterine cervix is a rare and highly malignant tumor, and its etiopathogenesis is strongly related to high-risk HPV infections.

**Methods:**

The clinicopathological data of 30 cases of cervical primary small cell carcinoma were retrospectively analyzed. In situ hybridization, polymerase chain reaction and reverse dot-blot hybridization were employed to detect HPV DNA in both small cell carcinoma and other coexisting epithelial tumors. Immunohistochemistry was used to detect the protein expression of p16 and p53.

**Results:**

Amongst 30 patients with cervical primary small cell carcinoma, 15 patients simultaneously exhibited other types of epithelial tumors, including squamous cell carcinoma, adenocarcinoma, squamous cell carcinoma in situ, and adenocarcinoma in situ. Most tumor cells infected with HPV presented integrated patterns in the nuclei by in situ hybridization. HPV DNA was detected in every small cell carcinoma case (100%) by polymerase chain reaction and reverse dot blot hybridization. 27 cases (90%) harbored type 18, and 15 (50%) displayed multiple HPV18 and 16 infections. The prevalence of HPV 18 infection in small cell carcinoma was higher than in cervical squamous and glandular epithelial neoplasms (*P* = 0.002). However, similar infection rates of HPV 16 were detected in both tumors (*P* = 0.383). Both small cell carcinoma and other types of epithelial tumors exhibited strong nuclear and cytoplasmic staining for p16 in all cases. Three cases of small cell carcinoma revealed completely negative p53 immunohistochemical expression in 15 cases of composite tumors, which suggested *TP53* nonsense mutation pattern. The pure small cell carcinoma of uterine cervix had similar mutation or wild type pattern for *TP53* compared with composite tumor (*P* = 0.224).

**Conclusions:**

Cervical small cell carcinomas are often associated with squamous or glandular epithelial tumors, which might result from multiple HPV infections, especially HPV 16 infection. Multiple HPV infections were not correlated with tumor stage, size, lymphovascular invasion, lymph node metastasis, or prognosis. Furthermore, careful observation of specimens is very important in finding little proportion of small cell carcinoma in the composite lesions, specifically in cervical biopsy specimens, in order to avoid the missed diagnosis of small cell carcinoma.

## Background

Numerous clinical and experimental studies have demonstrated a closed etiopathogenetic relationship between the development of cervical cancers (including squamous cell carcinoma, adenocarcinoma, and small cell carcinoma) and high-risk human papillomavirus (HPV) infection [1]. It has been implicated that squamous cell carcinoma and adenocarcinoma are correlated to HPV 16 infection, whilst cervical small cell carcinoma is linked to HPV 18 [2, 3]. As an uncommon and highly malignant tumor, small cell carcinoma of uterine cervix has similar morphological features to tumors arising in the lung. The clinicopathological features of cervical small cell carcinoma and its relationship with HPV infection has been widely studied, including case series and larger retrospective population-based reports [2, 4]. Although some studies report that cervical small cell carcinoma is often associated with other cervical cancer or intraepithelial neoplasia [5], the clinicopathological features of cervical composite tumors, including small cell carcinoma, were rarely characterized. In these studies, HPV infection was detected using a variety of methods [3, 6] such as immunohistochemistry, polymerase chain reaction (PCR), in situ hybridization (ISH), reverse dot blot hybridization (RDDH), and Southern blot hybridization. The inconsistent results affected the understanding of the relationship between HPV infection and cervical small cell carcinoma. Many studies revealed the preponderant infection of HPV 18 in cervical small cell carcinoma [2], but multiple infections were rarely reported.

In this report, the clinicopathological features of 30 patients were analyzed, including 15 patients with pure cervical small cell carcinoma, and 15 cases with composite cervical tumors composed of small cell carcinoma and other types of epithelial tumors. The HPV infection in these tumors was detected by ISH and PCR-RDDH, and the expression of p16 and p53 proteins were examined using immunohistochemistry. Our results demonstrate that cervical small cell carcinoma is often associated with squamous or glandular epithelial tumors, and these tumor cells usually exhibit overexpression of p16. Small cell carcinomas and adenocarcinomas or squamous cell carcinoma in situ showed all completely negative p53 immunohistochemical expression in three of 15 composite tumors of uterine cervix. To the best of our knowledge, this is the first study to report that multiple infections of HPV 18 and 16 developed in half of cervical small cell carcinomas. Furthermore, this is the first report to make the distinction that HPV 18 infection is closely correlated to the occurrence of cervical small cell carcinoma while HPV 16 infection is involved in cervical squamous cell carcinoma or adenocarcinoma in the co-existing tumor cases. Multiple infections of HPV subtypes were not related to tumor stage, size, lymphovascular invasion, lymph node metastasis, or prognosis. In addition, our study also confirmed for the first time that patients with composite tumors had similar HPV infection subtypes, clinicopathological features, and prognosis compared to patients with pure small cell carcinoma.

## Methods

### Case selection

Thirty cases of cervical primary small cell carcinoma were retrieved from the 2009–2017 surgical pathology archives of the Xijing Hospital. These included 23 hysterectomies, 1 conization, and 6 biopsies. To rule out the possibility of metastatic small cell carcinoma from the lung or other organs, the clinical data were carefully reviewed. Hematoxylin and eosin-stained slides of primary cervical neoplasm were re-examined to confirm the original diagnosis. Immunohistochemical staining for neuroendocrine markers, including Leu-19, synaptophysin, chromogranin, and neuron-specific enolase, was performed, and more than half of small cell carcinoma cells showed positive expression for two or more markers. The clinical records and follow-up of these patients were examined. This study received approval from the Ethics Committee of the Xijing Hospital.

### ISH analysis

Three-micron sections containing small cell carcinoma and other types of epithelial tumors were examined for HPV DNA by ISH staining with INFORM® HPV III Family 16 and 6 Probe (Ventana Medical Systems, Inc., Tucson, AZ, USA) for high-risk HPV types (16, 18, 31, 33, 35, 39, 45, 51, 52, 56, 58, and 66) and low-risk HPV types (6 and 11), respectively. The ISH assay was performed according to the manufacturer’s protocol using the Ventana BenchMark XT system (Ventana Medical Systems, Inc.). Labeling was detected with the ISH iVIEW™ Blue Plus Detection Kit (Ventana Medical Systems, Inc.). The positive and negative controls were carried out using HPV quality control slides provided by Ventana Medical Systems, Inc. The HPV signals were detected in the nuclei of tumor cells, and the signal patterns were categorized as either episomal staining pattern or punctuate staining pattern. The former presents as a homogeneous globular navy-blue to black signal in the entire nuclei of tumor cells, and the latter shows single or multiple sparsely distributed and dot-like navy-blue punctae in the nuclei of the tumor cells.

### DNA extraction and HPV detection using PCR-RDDH

Eight-micron sections were prepared from formalin-fixed, paraffin-embedded (FFPE) tissues of cervical tumor samples. Tumor tissues were dissected using sterile blades from slides and were collected into 1.5 ml Eppendorf tubes. Amongst 15 specimens with composite carcinomas, small cell carcinoma and other types of epithelial tumors were successfully isolated in seven specimens. 37 samples of tumor cells were collected, including 22 cases of small cell carcinoma, 8 cases of mixed tumors, 2 cases of squamous cell carcinoma, 2 cases of adenocarcinoma, 2 cases of squamous cell carcinoma in situ, and 1 case of adenocarcinoma in situ. DNA extraction was carried out using QIAamp® DNA FFPE Tissue Kit (Cat No. 56404, QIAGEN GmbH, Hilden, Germany) following the manufacturer’s guidelines. The samples were digested with proteinase K in a volume of 200 μL at 56 °C overnight, and 20 μL of DNA aliquot was obtained finally. The quality of the genomic DNA extracted was tested by agarose gel electrophoresis with ethidium bromide staining. For the PCR, 2 μL of DNA aliquot was used, and broad-spectrum HPV DNA amplification was performed using primers for GP5+/6+ (GP5+: 5’-TTTGTTACTGTGGTAGATACTAC-3′, GP6+: 5’-GAAAAATAAACTGTAAATCATATTC-3′) with a total reaction volume of 50 μL. PCR amplification was performed in the following conditions: 50 °C for 15 min, 95 °C for 10 min, 94 °C for 30 s, 46 °C for 90 s, 72 °C for 30 s, 40 cycles, 72 °C for 5 min. DNA RDDH was performed with the HPV Genotyping Detection Kit (Asia Energy Biotechnology Co, Ltd., Shenzhen, China) to simultaneously identify 23 HPV subtypes, including HPV 6, 11, 16, 18, 31, 33, 35, 39, 42, 43, 45, 51, 52, 53, 56, 58, 59, 66, 68, 73, 81, 82, and 83. PCR amplification products were boiled 10 min to obtain single-stranded DNA. Samples were then added in low density gene patches with a total of 23 gene probes of different HPV subtypes and hybridization was performed at 51 °C for 3 h. The gene patches were incubated in peroxidase solution for 30 min, and then developed in color reagent (19 ml 0.1 mol/L sodium citrate, 1 ml tetramethylbenzidine, and 2 μL 30% H_2_O_2_) for 60 min in the dark. HPV subtypes were determined by the positive point on the HPV genotype profile on the membrane. HPV-positive and negative controls were also included in every experiment.

### Immunohistochemistry

Immunohistochemistry was performed on 3 μm thick tissue sections using the Ventana BenchMark XT system (Ventana Medical Systems, Inc.). P16 (clone: 6H12) and p53 (clone: DO-7) antibodies were purchased from Maxin Corp. (Fuzhou, China) and DAKO Corp. (Carpinteria, CA), respectively. Immunohistochemical staining was conducted employing the Roche Ultraview DAB Detection Kit (Ventana Medical Systems, Inc.) following the manufacturer’s instructions. The positive and negative control slices were also run simultaneously. Nuclear/cytoplasmic staining was considered positive for p16, and p53 protein showed nuclear expression. A tumor was recorded positive for p16 if more than 50% of the tumor cells showed immunoreactivity. IHC for p53 includes mutation pattern and wild type pattern. Strong and diffuse nuclear staining or complete negative with internal control is considered to be mutation pattern, and focal and weak staining correlate with a wild type pattern of p53.

### Statistics

Statistical software SPSS17.0 (SPSS, Inc., Chicago, IL, USA) was employed in this report. Pearson Χ^2^ test or Fisher’s exact test was adopted for correlation analysis of enumeration data. Wilcoxon rank sum test was used for comparison of patients’ age and tumor size. P<0.05 was considered as a statistically significant.

## Results

### Clinicopathological features

Among 30 patients with cervical primary small cell carcinomas included in this study, 15 patients were diagnosed with pure cervical small cell carcinoma. In the remaining 15 cases of composite tumors, 7 cases also exhibited invasive cervical squamous cell carcinoma, 4 cases also showed cervical squamous cell carcinoma in situ, 3 cases also had cervical adenocarcinoma, and one patient also displayed cervical adenocarcinoma in situ. The age of all 30 patients at diagnosis ranged from 31 to 74 years (mean 46.4 years, median 41 years). The mean and median age of the 15 patients with cervical composite tumors was 46.4 and 45 years, respectively. 22 patients presented with abnormal vaginal bleeding or contact bleeding. Two patients showed no clinical symptoms, and the tumors were found following physical examination. Most of the tumors displayed exophytic growth with or without cervical erosion. The tumor sizes in the 24 hysterectomies and cervical conization specimens ranged from 1 to 6 cm (mean 3.0 cm, median 2.5 cm). 16 tumors (66.7%) were FIGO stage IB, 3 stage IIA, 2 stage IA, 1 stage IIB, 1 stage IIIA, and 1 stage IVB. Accurate staging information was not acquired for 6 cases of cervical biopsy specimens. 21 patients underwent radical hysterectomy with bilateral or partial adnexectomy and pelvic lymph node dissection. Two patients received radical hysterectomy with pelvic lymph node dissection, and one case underwent cervical conization owing to the superficial invasion depth of small cell carcinoma. Accurate surgical methods in 6 out-patients were not obtained in this study. 15 patients underwent postoperative chemotherapy combined with radiotherapy, and 6 patients received postoperative chemotherapy. Postoperative treatment strategies were not obtained in 9 other patients. Follow-up data of 16 cases was obtained: 7 patients died with a survival time ranging from 5 to 24 months (median survival time: 7 months). Survival time was more than two years in 4 out of 10 confirmed surviving patients, and two patients free of disease have undergone a follow-up at 67 and 88 months.

**Fig. 1:**
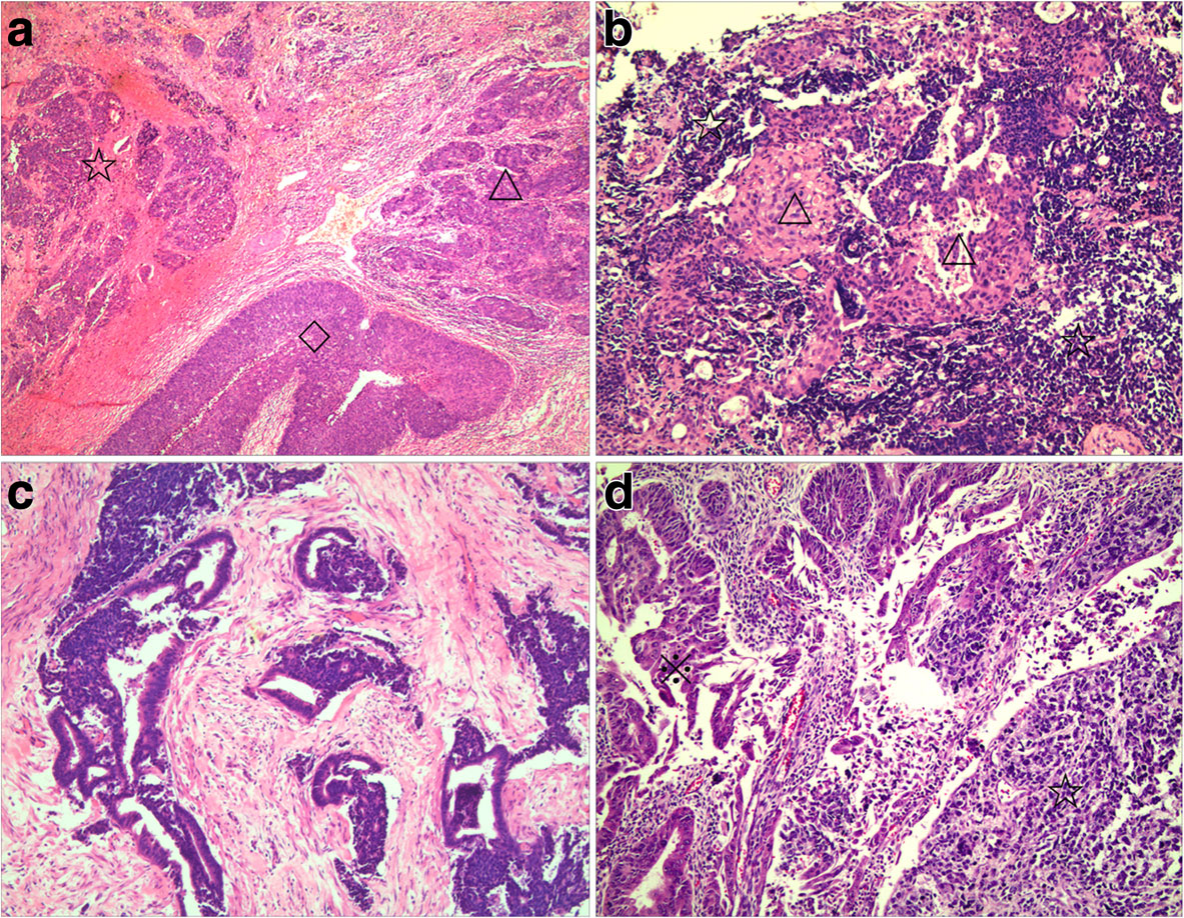
Cervical small cell carcinoma associated with other types of epithelial tumors (hematoxylin-eosin staining). **a** Squamous cell carcinoma (△) and squamous cell carcinoma in situ (◇) detected in the superficial parts of small cell carcinoma (☆) (× 40). **b** Squamous cell carcinoma (△) intermingled with small cell carcinoma (☆) (× 100). **c** Small cell carcinoma (☆) mixed with adenocarcinoma (※) (× 100). **d** Adenocarcinoma (※) present at the periphery of small cell carcinoma (☆) (× 100)

**Fig. 2:**
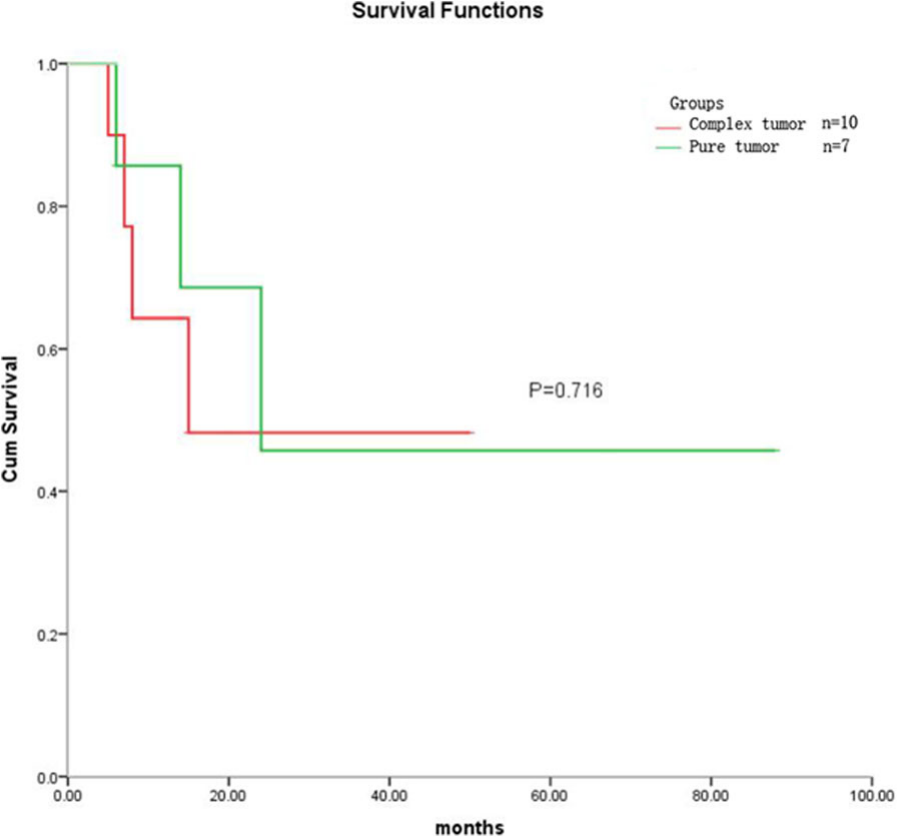
Survival analysis of 17 patients with follow-up data. The patients with composite cervical tumors had similar prognosis to patients with pure small cell carcinoma

Histologically, cervical primary small cell carcinomas showed morphological features similar to those seen in the pulmonary counterpart. Densely packed small tumor cells often formed a sheet-like diffuse growth pattern. Neuroendocrine growth patterns, such as orderly tubular, trabecular, organoid, and nuclear palisading patterns, were less illustrated. Tumor cells showed round, ovoid, or spindled nuclei and scant cytoplasm. Nuclear chromatin is finely granular, and nucleoli were absent or inconspicuous. Numerous mitotic figures and extensive necrosis were commonly observed. Furthermore, squamous and glandular epithelial neoplasms were observed in 15 cases. Most squamous cell carcinomas developed in the superficial parts of small cell carcinomas (Fig. 1a). However, in two cases, the squamous cell carcinoma intermingled with the small cell carcinoma (Fig. 1b). In three cases of cervical small cell carcinoma associated with adenocarcinoma, one case illustrated mixed small cell carcinoma and adenocarcinoma (Fig. 1c), and two patients revealed adenocarcinoma present at the periphery of the small cell carcinoma (Fig. 1d). Five cases of cervical small cell carcinoma associated with carcinoma in situ all illustrated adjacent relationships between small cell carcinoma and squamous cell carcinoma in situ or adenocarcinoma in situ. Although more composite tumors were found in hysterectomy specimens than in biopsy specimens in this study, the discovery of composite lesions was not related to specimen type (*P* = 0.651). There was no statistical difference in patients’ age, FIGO stage, lymph node metastasis, lymphovascular invasion, or prognosis between pure small cell carcinoma and composite tumors (Table 1, Fig. 2). Patients with cervical composite tumors had similar prognosis to patients with pure small cell carcinoma (*P* = 0.716). The prognosis of these patients was determined by the composition of small cell carcinoma. Six cases (26.1%) had regional nodal metastasis, including 5 cases of metastatic small cell carcinoma and one case of metastatic squamous cell carcinoma. One patient displayed small cell carcinoma involving vaginal stump, and ovarian metastatic small cell carcinoma was found in another patient. In the 24 patients who underwent hysterectomies or cervical conization, lymphovascular invasion of small cell carcinoma was detected inside or within the tumor in 21 cases, of which two patients had simultaneous lymphovascular invasion of squamous cell carcinoma. Only one case presented with lymphovascular invasion of small cell carcinoma in 6 cervical biopsy specimens. Lymphovascular invasion was more likely to be found in hysterectomy or conization specimens than in biopsy specimens (*P* = 0.002).

**Table 1:** Clinicopathological features of cervical small cell carcinoma with or without other types of epithelial tumors

	Pure small cell carcinoma	Small cell carcinoma associated with other epithelial tumors	*P* value
Cases (n)	15	15	
Specimen type			0.651
hysterectomy or cervical conization	11	13	
biopsy	4	2	
Age (years)	46.4 (34–68)	46.4 (31–74)	0.967
Size of tumors	3.4 (2–6)	2.5 (1–4.5)	0.105
FIGO stage			0.605
I	9	9	
II	2	2	
III		1	
IV		1	
Lymph node metastasis			1.000
Yes	3	3	
No	8	9	
Lymphovascular invasion			1.000
Yes	11	11	
No	4	4

### HPV DNA detection and typing

**Fig. 3:**
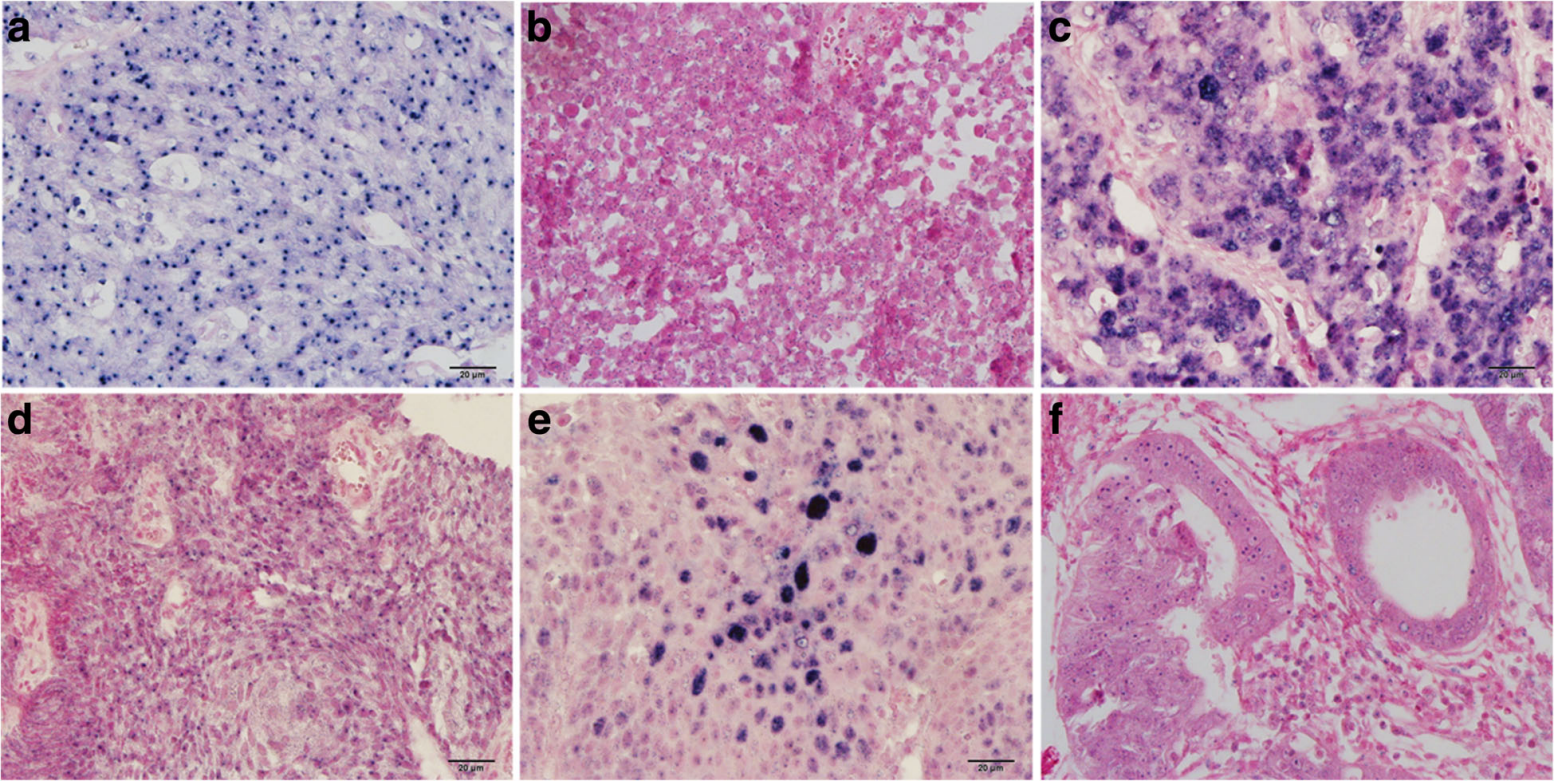
HPV DNA detection using in situ hybridization (× 400). **a** Small cell carcinoma illustrated punctate staining pattern with one navy-blue puncta signal in the nuclei. **b** Multiple small puncta inside the nuclei of small cell carcinoma represents low viral copy number of HPV DNA. **c** Diffuse small particles were observed in the nuclei of small cell carcinoma. **d** Squamous cell carcinoma displayed punctuate signal patterns. **e** Squamous cell carcinoma illustrated episomal staining pattern. **f** Tumor cells of adenocarcinoma positive with one navy-blue puncta

ISH of tissue sections revealed that most specimens were positive for the INFORM HPV III Family 16 Probe in both small cell carcinomas and other types of epithelial tumors. 29 out of 30 cases of small cell carcinomas showed positive staining, including 21 cases with only one navy-blue to black puncta (Fig. 3a), 6 cases with multiple small blue puncta inside the nuclei (Fig. 3b), and 2 cases with multiple puncta-like staining to diffuse small particles (Fig. 3c). These staining signals represent viral integrative patterns in 27 cases. In some cases, the nuclear viral integrative pattern was so discrete that microscopic examination at a higher magnification (40× objective) was required to judge nuclear epithelial cell localization, which represented very low viral copy number. Two cases of small cell carcinoma showed both episomal and integrative staining patterns. In 7 cases of cervical squamous cell carcinoma, 5 cases illustrated punctate signals in the nuclei of tumor cells (Fig. 3d) and 2 cases displayed episomal staining patterns. Two out of four cases of squamous cell carcinoma in situ revealed episomal staining patterns (Fig. 3e), and all tumor cells of cervical adenocarcinoma and adenocarcinoma in situ illustrated punctate staining (Fig. 3f). Interestingly, one case displayed positive staining for small cell carcinoma with punctate pattern but negative for squamous cell carcinoma, and another was positive for cervical adenocarcinoma in situ with punctate staining, but negative for small cell carcinoma. The INFORM HPV III Family 6 Probe was employed to detect low-risk HPV genotypes, and no positive tumor cells were observed in both small cell carcinoma and other types of epithelial tumors.

**Fig. 4:**
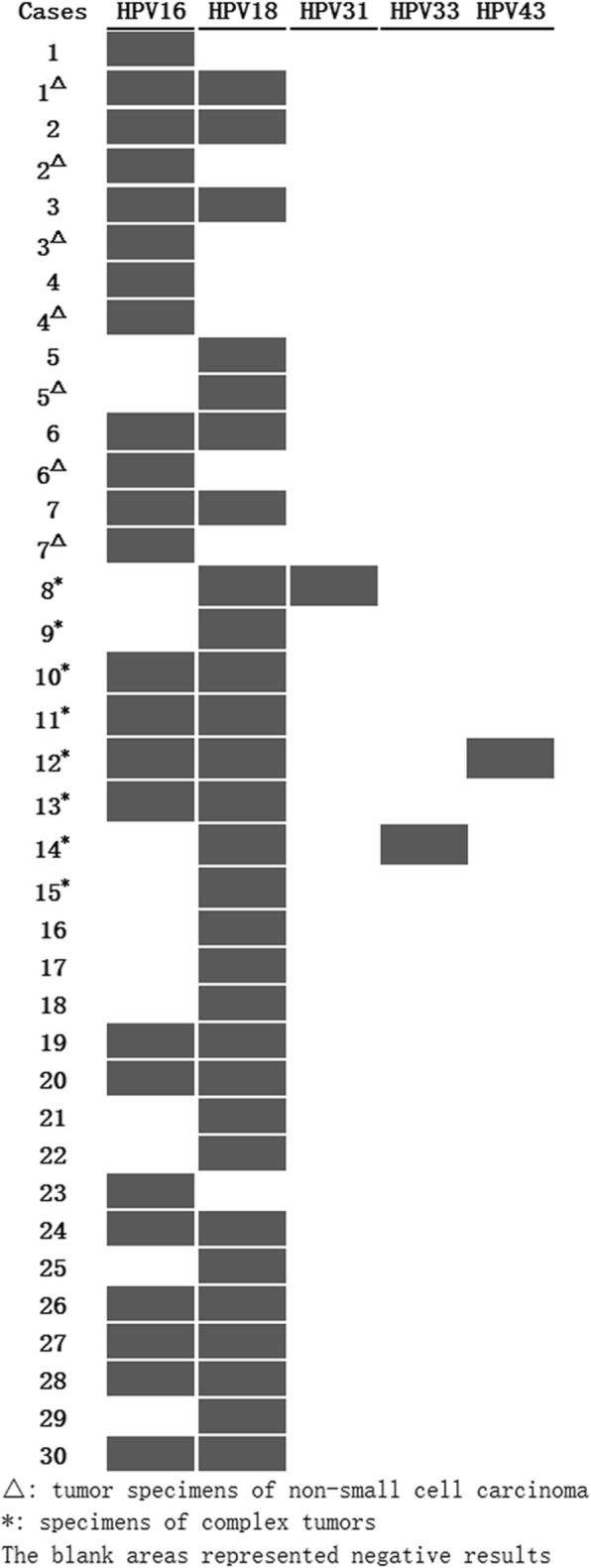
HPV typing using polymerase chain reaction-reverse dot blot hybridization

Thirty-seven specimens of cervical small cell carcinoma and other types of epithelial tumors were tested for HPV DNA by PCR-RDDH, and all were positive (Fig. 4). The prevalence of HPV 18 and 16 was 78.4% (29/37) and 64.9% (24/37), respectively. 48.6% (18/37) of specimens demonstrated multiple infections with various HPV subtypes, and the predominant multiple infections were HPV 16 and 18 at a rate of 43.2% (16/37). The rare infection of HPV 31, 33, and 43 was detected in three cases of small cell carcinoma with multiple HPV infections. The prevalence of HPV 18 and 16 was 90.0% (27/30) and 60.0% (18/30) in the 30 cases of small cell carcinoma, respectively. Multiple infections were found in 17 cases of small cell carcinoma, among which coinfection of HPV 18 and 16 was detected in 15 cases. Multiple infections in composite tumors (73.3%) were more common than in pure small cell carcinoma (46.7%), but there was no statistical difference (*P* = 0.136). The infection prevalence of HPV 16 and 18 was not significantly different between the composite tumor group and the pure tumor group (*P* = 0.456 and 1.00, respectively). The HPV DNA subtypes in different tumor components were detected in seven cases of composite tumors, and the consistent HPV type was detected in two cases with single infection. Four cases showed that tumor cells of small cell carcinoma were positive for HPV both 16 and 18, while other types of epithelial tumors, including adenocarcinoma, squamous cell carcinoma, and squamous cell carcinoma in situ, only displayed HPV 16 infection. The infection rate of HPV 18 in small cell carcinoma was significantly higher than in other types of epithelial tumors (*P* = 0.008). However, similar infection rates of HPV 16 were measured in both groups (*P* = 0.382, Table 2). In addition, multiple infection with HPV subtypes was not related to tumor stage, size, lymphovascular invasion, lymph node metastasis, or prognosis (*P* = 0.187, 1.00, 1.00, 0.179, and 0.498, respectively).

**Table 2:** HPV infection in small cell carcinoma and other types of epithelial tumors

	Small cell carcinomas	Squamous or glandular epithelial neoplasms	*P* value
Cases (n)	22	7	
HPV 18	19	2	0.008
HPV 16	14	6	0.382
HPV 18 + HPV 16	11	1	0.187

### Immunohistochemical expression of p16 and p53

**Fig. 5:**
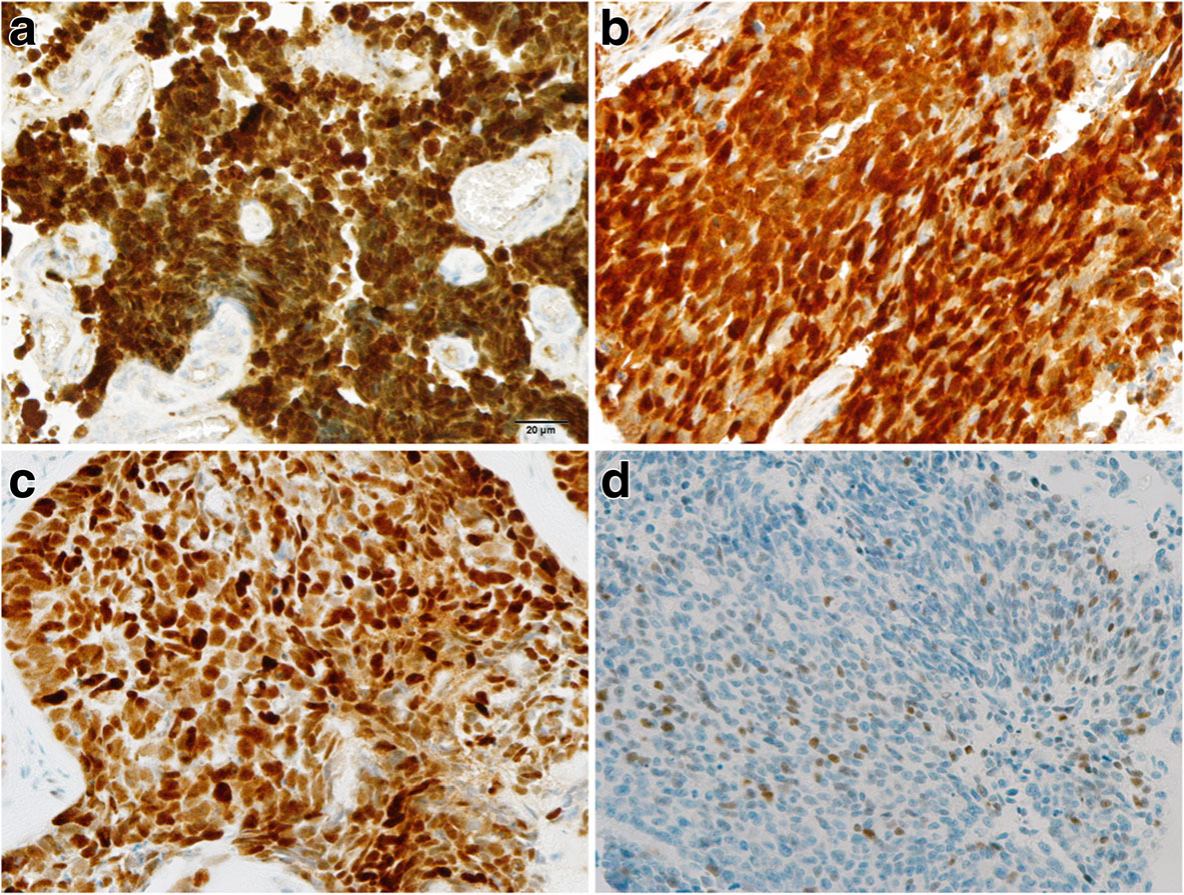
Immunohistochemical expression of p16 and p53 (× 400). Strong and diffuse nuclear and cytoplasmic staining for p16 was noted in cervical small cell carcinoma (**a**) and squamous cell carcinoma (**b**). **c** Nuclear staining for p53 was observed in cervical squamous cell carcinoma, wich revealed *TP53* missense mutation. **d** Cervical small cell carcinoma illustrating scattered, weakly positive nuclear staining for p53 revealed wild type pattern of *TP53*

Strong and diffuse nuclear and cytoplasmic staining for p16 protein was noted in all cases of cervical small cell carcinoma (Fig. 5a), squamous cell carcinoma (Fig. 5b), squamous cell carcinoma in situ, adenocarcinoma, and adenocarcinoma in situ. In contrast, the nonneoplastic squamous and glandular epithelia were either negative or showed focal and weak cytoplasmic positivity. Strong and diffuse nuclear staining for p53 protein was observed in one case of cervical squamous cell carcinoma (Fig. 5c) and in one case of squamous cell carcinoma in situ, which revealed *TP53* missense mutation. Scattered tumor cells illustrated weak or moderate positivity for p53 with rates ranging from 1 to 60% (Fig. 5d), which meant wild type pattern of *TP53*. Three cases of composite tumors showed negative staining both in small cell carcinoma and in adenocarcinoma or squamous cell carcinoma in situ, which suggested *TP53* nonsense mutation. The pure small cell carcinoma of uterine cervix had similar mutation or wild type pattern of *TP53* compared with composite tumor (*P* = 0.224), and there was no difference between *TP53* mutation in small cell carcinoma and those in other epithelial neoplasms of uterine cervix (*P* = 0.682).

## Discussion

Small cell carcinoma of the uterine cervix is a rare and highly malignant tumor. The etiopathogenetic association between cervical small cell carcinoma and high-risk HPV infections has been well documented in some studies [1, 2, 7]. Our study extend these findings by demonstrating that all 30 cases of cervical small cell carcinomas are related to high-risk HPV types 18 and 16, with predominant HPV 18 infection and multiple infections with HPV 18 and 16. In addition, a high frequency of cervical small cell carcinomas associated with other epithelial tumors was reported. This may be related with the common etiopathogenesis—HPV 18 and HPV 16. However, the prevalence of HPV 18 was different in cervical small cell carcinomas from squamous or glandular epithelial neoplasm.

The mean age at diagnosis for women with cervical small cell carcinoma was between 45 and 50 years [2], which is consistent with that of cervical squamous cell carcinoma. In this study, the mean age of the patients with cervical small cell carcinoma was 46.4 years. The patients had no specific clinical manifestations. Most cases presented with abnormal vaginal bleeding or contact bleeding. Exophytic growth was not different from other uterine cervical carcinomas. Of note, most of the patients in our study were in the early stage, which differs from previous studies [2, 4]. However, high rates of lymph node metastasis and poor prognosis were still observed in these stage I and II patients.

Primary small cell carcinomas of uterine cervix often coexist with squamous cell carcinomas or adenocarcinomas. Wang et al. reported that in 22 cases of primary cervical small cell carcinomas, two exhibited concordant high-grade squamous intraepithelial neoplasia and adenocarcinoma in situ [8]. In the study by Abeler et al., 12 of the 26 patients with cervical small cell carcinomas were associated with other forms of carcinoma, including squamous cell carcinoma (*n* = 6), adenocarcinoma (*n* = 5), and adenocarcinoma in situ (*n* = 1) [9]. Ishida et al. reported 10 cases of cervical small cell carcinoma, 7 of which were mixed with adenocarcinoma and/or squamous cell carcinoma, or cervical intraepithelial neoplasia [10]. Emerson et al. reported that in 19 cases of cervical small cell carcinoma, 6 cases were associated with adenocarcinoma, and three patients also had adenosquamous carcinoma, squamous cell carcinoma in situ, and adenocarcinoma [5]. In this study, 15 patients also displayed squamous or glandular epithelial neoplasms, including squamous cell carcinoma (*n* = 7), adenocarcinoma (*n* = 3), squamous cell carcinoma in situ (*n* = 4), and adenocarcinoma in situ (n = 1). Our results demonstrate a high frequency of cervical small cell carcinomas associated with squamous or glandular epithelial tumors. They had similar age composition and clinical manifestations as the patients of single cervical tumor. Chan et al. found that pure, rather than mixed histological pattern was a poor prognostic factor for survival [11]. However, in our study, patients of cervical small cell carcinoma with and without other types of epithelial neoplasms had similar prognosis, which was significantly worse than that of cervical squamous cell carcinoma and adenocarcinoma [12]. Therefore, the prognosis of patients with composite cervical cancer was determined by the composition of small cell carcinoma. In addition, in the case of biopsy specimens suspected of cervical epithelial disease, careful observation should be made to avoid the omission of small cell carcinoma. In this study, more lymphovascular invasion was observed in hysterectomy and cervical conization specimens. Therefore, obtaining as many specimens as possible improves the accuracy of the diagnosis, and it will more accurately estimate the prognosis.

High-risk HPV has been implicated in the carcinogenesis of cervical small cell carcinomas [1, 7]. A meta-analysis including more than 30,000 invasive cervical cancers revealed that HPV 16 (59%), 18 (13%), 58 (5%), 33 (5%), and 45 (4%) were the most prevalent subtypes in cervical squamous cell carcinomas. HPV 18 (37%), 16 (36%), 45 (5%), 31 (2%), and 33 (2%) were the most prevalent in cervical adenocarcinomas [13]. Many studies have established that the prevalence of different high-risk HPV types in cervical small cell carcinoma ranged from 50 to 100%, and that HPV 18 may be the most prevalent type [2]. Wang et al. reported that HPV18 and 16 were detected in 77.3 and 18.2% cases of cervical small cell carcinoma, respectively, and one case displayed HPV 18 and 16 co-infection [8]. Research by Abeler et al. demonstrated that HPV-18 is predominant in pure small cell carcinomas and in tumors with adenocarcinomatous areas, and that HPV-16 is found in pure small cell carcinomas and in tumors with areas of squamous cell differentiation [9]. In a study by Ishida et al., HPV 18 was detected in both small cell carcinomas and adenocarcinomatous components, and no other types of HPV were detected [10]. In our study, preponderant infection with HPV 18 was found in 27 of 30 cases of cervical small cell carcinoma. In the 15 cases of composite tumors, HPV 18 infection was more common in small cell carcinoma than in any other type of epithelial tumors. These findings are in line with the notion that HPV 18 infection is involved in the development of cervical small cell carcinoma and HPV 16 infection promotes the occurrence of cervical squamous cell carcinoma and adenocarcinoma.

Multiple infections were observed in more than half of the cervical small cell carcinomas in our study. Only one case of multiple infections was found in 7 cases of squamous and glandular epithelial neoplasm. Multiple infections of HPV 18 and 16, rather than pure HPV 18 infection, might play an important role in the pathogenesis of cervical small cell carcinoma. In previous studies, very few cases of multiple infections were reported [8]. One explanation for this might be found in the different detection methods or the population differences. In the research by Zhou et al., using HPV DNA detection by PCR-RDDH, multiple HPV DNA prevalence was 4.5% in 2452 women whom volunteered for cervical cancer screening in Shanghai, China [14]. A similar multiple infection prevalence rate was measured in 9012 women whom attended cervical cancer screening in Taihu River Basin, China [15]. In addition, reported HPV infection rates are closely related to the detection methods used. For example, in our study, the ISH assessment for HPV infection with low copy number was very difficult, displaying the lower sensitivity using ISH detection compared to PCR. Similar results were reported by Masumoto using ISH and direct sequencing of PCR products [16]. The prevalence of infection by multiple HPV genotypes was 20% in patients with cervical squamous cell carcinoma and adenocarcinoma [17]. One case of multiple HPV infection was confirmed in seven cases of squamous or glandular epithelial neoplasms in our study. Furthermore, the prevalence of multiple infections in small cell carcinoma was not significantly different in pure small cell carcinoma compared to the composite tumors, and multiple HPV infections did not affect the prognosis of these patients.

Overexpression of p16 has been well documented in high-risk HPV-related cervical squamous cell carcinomas and adenocarcinomas, as well as their precursor lesions [18, 19]. Many recent studies have shown that cervical small cell carcinoma also overexpressed p16 protein [20, 21]. In this study, we detected simultaneous overexpression of p16 in both small cell carcinoma and other types of epithelial tumors by immunohistochemistry. Although the overexpression of p16 was correlated with HPV18 and 16 infections in both pure tumors and composite tumors, it does not confirm that high-risk HPV infections result in the overexpression of p16. Indeed, small cell carcinoma negative for HPV DNA in the lung, colorectum, bladder, and ovaries also overexpress p16 [8, 20]. In our study, the mutation or wild type pattern of TP53 in small cell carcinoma was not significantly different between pure and composite tumors. Three patients with composite tumors showed completely negative p53 protein expression both in small cell carcinoma and in other epithelial tumors. Furthermore, two cases revealed strong and diffuse positive expression of p53 only in squamous cell carcinoma or squamous cell carcinoma in situ, while small cell carcinoma components of the same patients showed wild type pattern. These observations indicated TP53 mutation might involved in the occurrence of small cell carcinoma as in squamous cell carcinoma and adenocarcinoma of uterine cervix, but the pathogenesis of small cell carcinoma was not completely same to those of squamous cell carcinoma or adenocarcinoma.

Since only a single-center retrospective case series was studied, and limited cases were employed, this study should be repeated on a larger scale to confirm our findings. Furthermore, some patients did not complete their follow-up, thus the comprehensive and effective data were not obtained.

## Conclusions

In summary, our study demonstrated that cervical small cell carcinomas closely correlate with HPV18 and HPV 16 infections. The patients of cervical small cell carcinomas with multiple infection of high-risk HPV may also promote the development of squamous or glandular epithelial neoplasms. In patients with composite cervical neoplasms, multiple infections of HPV 18 and 16 were involved in the development of cervical small cell carcinoma, while the occurrence of cervical squamous cell carcinoma or adenocarcinoma was closely related to HPV 16 infection. Multiple high-risk HPV infection was not related to tumor stage, size, lymphovascular invasion, lymph node metastasis, or prognosis in cervical small cell carcinoma. Similar HPV DNA genotypes, clinicopathological features, and prognosis were observed both in patients with pure small cell carcinomas and those with composite cervical tumors. The small cell carcinoma determined the prognosis of patients with cervical composite tumors. Because of the frequent presence of co-existing tumors, it is important to carefully examine the cervical biopsy specimens in surgical pathological examination.

## Abbreviations

FIGO: International Federation of Gynecology and Obstetrics
HPV: Human papillomavirus
ISH: In Situ Hybridization
PCR: Polymerase chain reaction
RDDH: Reverse dot blot hybridization
